# Cerebral blood flow changes induced by high-frequency repetitive transcranial magnetic stimulation combined with cognitive training in Alzheimer's disease

**DOI:** 10.3389/fneur.2023.1037864

**Published:** 2023-01-24

**Authors:** Yuanyuan Qin, Li Ba, Fengxia Zhang, Si Jian, Min Zhang, Wenzhen Zhu

**Affiliations:** ^1^Department of Radiology, Tongji Hospital, Tongji Medical College, Huazhong University of Science and Technology, Wuhan, Hubei, China; ^2^Department of Neurology, Tongji Hospital, Tongji Medical College, Huazhong University of Science and Technology, Wuhan, Hubei, China; ^3^Department of Rehabilitation, RenMin Hospital of Wuhan University, Wuhan, Hubei, China

**Keywords:** Alzheimer's disease, repetitive transcranial magnetic stimulation, arterial spin-labeling, cerebral blood flow, precuneus, posterior cingulate cortex

## Abstract

**Background and purpose:**

Hypoperfusion of the posterior cingulate cortex (PCC) and precuneus has consistently been reported in patients with Alzheimer's disease (AD). Repetitive transcranial magnetic stimulation (rTMS) combined with cognitive training (COG) is effective in alleviating the symptoms of patients with mild AD. This study investigated the effects of rTMS-COG therapy on cerebral blood flow (CBF), with a special interest in the PCC/precuneus, and whether observed CBF changes are associated with changes in neuropsychological assessments in AD.

**Materials and methods:**

Twenty-one patients with mild or moderate AD were randomly divided into real rTMS (*n* = 11) and sham treatment (*n* = 10) groups, both combined with COG. Neuro-navigated 10 Hz rTMS was used to stimulate the left dorsolateral prefrontal cortex (DLPFC) and then the left lateral temporal lobe (LTL) for 20 min each day for 4 weeks in the real rTMS group. All patients with AD underwent neuropsychological assessment, pseudo-continuous arterial spin labeling, and structural 3D T1-weighted MRI before treatment (T0), immediately after treatment (T1), and 4 weeks after treatment (T2). CBF in the precuneus, PCC, and stimulation targets at the region-of-interest (ROI) level, as well as whole-brain CBF changes at the voxel level, were compared between the two groups at three timepoints.

**Results:**

rTMS-COG therapy revealed significant group × time interactions for the Mini-Mental State Examination (F = 5.339, *p* = 0.023, η^2^ = 0.433) and activities of daily living (F = 5.409, *p* = 0.039, η^2^ = 0.436) scores. The regional CBF in the precuneus showed a significant group × time interaction (F = 5.833, *p* = 0.027, η^2^ = 0.593). For voxel-level analysis, a significant group main effect was found in the left limbic lobe cluster, with the maximal peak in the left parahippocampus (*p* < 0.001, uncorrected, peak at [−16 −8 −24]). Simple effects analysis indicated that rTMS-COG therapy induced a decrease in CBF in the precuneus at T1 (*p* = 0.007) and an increase in the left parahippocampus at T2 (*p*=0.008). CBF decrease in the precuneus was correlated with better cognitive function immediately after treatment (T1) (*r* =−0.732, *p*=0.025).

**Conclusion:**

Neuropsychological assessments showed immediate and long-term effects on cognitive function and activities of daily living after rTMS-COG therapy. CBF changes induced by high-frequency rTMS-COG therapy are region-dependent, showing immediate effects in the precuneus and long-term effects in the left parahippocampus. These results provide imaging evidence to understand the underlying neurobiological mechanism for the application of rTMS-COG in AD.

## Introduction

Alzheimer's disease (AD) is a neurodegenerative disease characterized by gradual cognitive impairment. It is the most common form of dementia and imposes a great burden on both patients and their caregivers. Although drugs such as acetylcholinesterase inhibitors are commonly used in clinical practice, they only delay the disease course to a certain extent and cause adverse side effects (1). Repetitive transcranial magnetic stimulation (rTMS) is a non-invasive neuromodulation technique that has been shown to induce short (“on-line”) and long (“off-line”) effects in different ways (2). The application of magnetic pulses to a target location in the brain repetitively generates long-lasting beneficial effects persisting for several weeks or even months after treatment (3–6). rTMS has been approved by regulatory agencies as a therapeutic tool for a number of neuropsychiatric disorders, such as drug-resistant depression and obsessive-compulsive disorder (7, 8). However, the application of rTMS in AD is limited, partly because of the inconsistency in stimulation parameters and scarcity of in-depth research on the underlying neurobiological mechanism.

Recent studies have shown that rTMS can ameliorate the symptoms of AD and has potential as a therapeutic method for mild-to-moderate AD (3, 4). In patients with AD and those with mild cognitive impairment (MCI), rTMS has shown favorable outcomes in cognitive function (9, 10), activities of daily living (ADL) (11), attention and psychomotor speed (12, 13), behavioral and psychological symptoms (14), and language performance (15, 16). Interlaced with cognitive training (GOG), rTMS has additional beneficial effects, possibly as good as cholinesterase inhibitors (17). Although rTMS has been gradually applied in the field of neurodegenerative diseases, its related neurochemical and functional changes in the brain remain poorly understood.

The site of stimulation, frequency of stimulation, and number of sessions are highly relevant to the therapeutic effect achieved by rTMS. The dorsal lateral prefrontal cortex (DLPFC) is the most commonly selected site for magnetic stimulation in patients with AD (11, 15, 17) and MCI (18, 19). Other regions, including the precuneus (20, 21), lateral parietal cortex (22), right inferior frontal gyrus, right superior temporal (12), and left lateral temporal lobe (LTL) (23) have also been reported. High-frequency rTMS (>5 Hz) is usually more appropriate than low-frequency rTMS (<1 Hz) for the treatment of AD (4, 11, 24). Long-term (≥5 sessions) and multiple-site stimulation produces better cognitive improvement than short-term (≤3 sessions) and single-target rTMS treatment (3). Therefore, by stimulating the left DLPFC and LTL with high-frequency and long-term rTMS, our previous study revealed a significant increase in the concentration of N-acetylaspartate in the left DLPFC in patients with AD (23), reflecting better neuronal function after treatment. rTMS influences not only the stimulation targets but also distant sites that are anatomically or functionally connected to the stimulated region of the brain (19, 25).

Quantitative perfusion analyses of the brain are extremely useful for investigating the neurobiological changes induced by rTMS. Arterial spin labeling (ASL) is particularly attractive because it avoids the need for exogenous contrast agents. Using arterial blood water as a freely diffusible endogenous tracer, the ASL technique enables non-invasive and repeated measurement of cerebral blood flow (CBF) (26). Although several brain regions have been shown to be involved in MCI-AD, the posterior cingulate cortex (PCC) and precuneus are among the most consistent regions of hypoperfusion (27–29). Tau retention and astrogliosis/neurodegeneration in the PCC/precuneus play important roles in determining the dementia state in early AD (30). As the major nodes within the default mode network (DMN) (31), the PCC/precuneus has a wide connection with other regions of the brain, including the prefrontal cortex and temporal lobe (32). Our previous study revealed metabolic changes in the stimulation targets; however, CBF changes induced by rTMS-COG therapy have not yet been clarified. In this study, CBF changes in the stimulation targets and PCC/precuneus were first analyzed at the region-of-interest (ROI) level. Exploratory voxel-wise analysis was then conducted on the whole brain. The purpose of this study was to explore (1) the effect of rTMS-COG therapy on regional CBF changes, with a special interest in the PCC/precuneus, and (2) whether the observed CBF changes are associated with cognitive function alterations.

## Materials and methods

### Ethical approval and patient consent

This randomized, double-blind, sham-controlled study was approved by the institutional review board of Tongji Hospital in Wuhan, China (Clinical Trial Registry registration number: ChiCTR-INR-16009227). Written informed consent was obtained from all participants or their caregivers.

### Participants and experiment design

Patients with mild or moderate AD were enrolled with the following inclusion criteria: (1) age >50 years and right-handed; (2) more than 6 years of education; (3) met the criteria for probable AD based on NINCDS-ADRDA (33); (4) Clinical Dementia Rating (CDR) scale memory score of 0.5–2; (5) Hachinski Ischemia Score of <4; and (6) stable anti-dementia medication regimen (donepezil or memantine) for at least 3 months. Real rTMS or sham stimulation was repeated five times per week (from Monday to Friday) for 4 weeks. Cognitive training for up to 1 h was performed in conjunction with rTMS every day. Each patient with AD underwent neuropsychological assessment and MRI before treatment (T0), on Saturday morning at the end of 4 weeks of treatment (T1), and 4 weeks after treatment (T2). Patients with MRI or rTMS contraindications, as well as those with other significant neurological or psychiatric diseases, potential cerebrovascular disease, and brain tumors detected on structural MRI before treatment, were excluded.

### rTMS and cognitive training protocol

The left DLPFC and left LTL were chosen as stimulation targets, guided by marked coordinates *via* an optical navigation system (Magventure stimulator, Denmark and Localite TMS Navigator, Germany). It provides an electromagnetic stimulation coil in combination with a neuronavigation unit, allowing 3D tracking of the coil and simultaneous visualization of all stimulation sites. The electromagnetic stimulation coil was initially positioned over the left DLPFC and then over the LTL. The Talairach coordinates of the DLPFC were X = −35, Y = 24, and Z = 48 at approximately halfway between F3 and F7, and the Talairach coordinates of the LTL were X = −60, Y = −15, and Z = −15 at ~T3, using the 10/20 EEG system. Real rTMS stimulation was applied using a butterfly-shaped coil (MCF-B65, inner diameter = 35 mm, outer diameter = 75 mm, winding height = 12 mm) with the following parameters: repetition 10 Hz with each train lasting for 5 s, intermittent for 25 s, and 20 trains with 1,000 pulses in total. Therefore, for each stimulation site, the rTMS operation lasted for ~10 min. The rTMS intensity was set at 100% of the patient's resting motor threshold (RMT). RMT was defined as the minimum intensity exerted by single-pulse TMS over the optimal area for stimulating the thumb with a visual twitch (hot spot) that elicited a minimum of 50 mV of motor evoked potential in 5 out of 10 trials over the contralateral first dorsal interosseous muscle (34). Sham stimulation was implemented with the same coil over the same scalp position but oriented with the front edge touching the scalp at 90°.

Cognitive tasks were selected by an experienced cognitive therapist and completed on an iPad tablet (version 9.1; Apple, USA). In rTMS-COG therapy, cognitive tasks are usually performed between rTMS trains (35). In our study, the memory tasks were completed during the whole process of rTMS stimulation for 20 min in total, whereas the others such as attention tasks, mathematical calculations, agility drills, language tasks, and logical thinking tasks were practiced sequentially after stimulation ceased, lasting ~40 min. The memory tasks were related to the activation of stimulation targets, whereas the other cognitive tasks were chosen depending on the specific domain of cognitive impairment, with each task lasting for 10 min. All cognitive tasks were completed by the patients on the same Apple tablet touch screen in a similar manner, guided by the same experienced therapist with no additional maintenance sessions. The details of the cognitive training were the same as those described in our previous study in detail (23).

Before administering TMS, a screening questionnaire was requested (36). Before and after each TMS session, the patient's blood pressure and heart rate were measured to ensure that the patient was in good condition before leaving.

### Neuropsychological assessments

Demographic and clinical characteristics, specifically sex, age, education level, disease duration, APOE gene type, CDR scale, Mini-Mental State Examination (MMSE), Alzheimer's Disease Assessment Scale-Cognitive Subscale (ADAS-cog) score, Addenbrooke's Cognitive Examination III (ACE-III), auditory verbal learning test (AVLT), Neuropsychiatric Inventory (NPI), and ADL were evaluated by a neurologist with more than 20 years of experience who was blinded to the group information of all patients. Higher MMSE, AVLT, and ACE-III scores, as well as lower ADAS-cog scores, indicate better cognitive function. Lower NPI and ADL scores indicate better conditions.

### MRI

All participants underwent MRI on a 3.0T MR scanner (Discovery MR 750, GE Healthcare, Chicago, IL, USA) with a 32-channel phased-array coil. 3D pseudo-continuous ASL (3D-pCASL) was performed at TR/TE = 4,788/14.6 ms, post label delay (PLD) = 1,525 ms, number of excitations (NEX) = 3, bandwidth = 62.5 kHz, readout of 8 arms × 1,024 samples, FOV = 240 × 240 mm, slice thickness/gap = 4/0 mm, and 34 pairs of labeled and control images. For co-registration of each individual's imaging data into a common space for processing and analysis, structural 3D T1-weighted images were acquired using brain volume (BRAVO) sequence, with repetition time/echo time (TR/TE) = 8.2/3.2 ms, inversion time = 450 ms, FOV = 240 × 240 mm, matrix = 256 × 256, NEX = 1, slice thickness = 1 mm, and 184 sagittal slices.

### MRI data processing and analysis

A cloud-based tool (ASL-MRICloud) was used for data processing (https://mricloud.org/) (37). ASL-MRICloud is integrated with T1-based brain segmentation and normalization tools to allow the generation of CBF maps in the Montreal-Neurologic-Institute (MNI) template space as well as CBF values at the ROI level. First, we uploaded the 3D-T1 BRAVO images using an elderly brain atlas set for T1-MultiAtlas analysis (38). ASL data and the.zip files from T1-MultiAtlas analysis were subsequently uploaded through the ASL MRI processing pipeline to provide regional CBF values. Quantification of CBF followed the kinetic model described in the ASL white paper (39), based on a T1 relaxation time of blood at 3.0T of ~1,600 ms, labeling efficiency of 0.8, brain–blood-partition coefficient of 0.9, labeling duration of ~1,500 ms, and post-labeling delay time of 1,525 ms. The image quality (from 1 indicating excellent to 4 indicating poor quality) was also reported in the results.

The regional CBF values of the PCC and precuneus, as well as the stimulation targets (left DLPFC and left LTL), were chosen for ROI-based analysis. For voxel-level analysis, the normalized CBF maps in the MNI template space were first smoothed using an 8-mm full width at half maximum Gaussian Kernel. Then exploratory voxel-wise analysis was conducted using a 2 × 3 flexible factorial design installed in SPM12 (https://www.fil.ion.ucl.ac.uk/spm/) with “group” (2 levels: real and sham) as between factor and “time” (3 levels: T0, T1, T2) as within-factor, with sex, age, education level, CDR and disease duration as covariates. The average CBF values within clusters that had significant main or interaction effects were extracted for further simple effect and correlation analyses.

### Statistics

The Shapiro–Wilk test was used to test the normal distribution of the data. Clinical characteristics at baseline were analyzed using a two-sample *t*-test or chi-square test. The neuropsychological assessments and CBF changes at the ROI level were analyzed using a mixed-design analysis of variance with “group” as between-subject factor and “time” as within-subject factor using SPSS software (version 21.0, USA). *Post-hoc* analysis was conducted in cases of a significant main effect or interaction effect using least significance difference correction. Sex, age, education level, CDR, and disease duration were set as covariates in the above tests. Pearson correlations between CBF changes and neuropsychological score changes (MMSE, ADAS-cog, ACE-III, AVLT, and ADL) were calculated in the real rTMS-COG therapy group. *p* < 0.05 was considered to indicate statistical significance.

## Results

### Baseline characteristics

All enrolled patients with AD received either real rTMS (*n* = 11) or sham treatment (*n* = 10), combined with cognitive training. Two patients in the sham group withdrew after their first revisit. One patient in the real group and two in the sham group were excluded because of incomplete MRI or neuropsychological examination at T2. All data were normally distributed, except for the NPI score, which was excluded from the statistical analysis. The real and sham treatment groups showed no significant differences in age, sex, education level, CDR, disease duration, MMSE, ADAS-cog, ACE-III, AVLT, and ADL at baseline (*p* > 0.05). The APOE gene type also showed no significant difference (*p* > 0.05). Clinical information of all participants at baseline is shown in Table 1.

**Table 1:** Demographic data of the participants at baseline.

	**Real rTMS-CT**	**Sham rTMS-CT**	***P*-value**
Sex (F/M)	8/2	3/3	0.611^a^
Age (years)	65.60 ± 8.06	66.50 ± 9.40	0.842^b^
Education (years)	11.80 ± 2.53	11.83 ± 3.13	0.983^b^
APOE (ε3/ε4, ε4/ε4, ε3/ε3)	5,1,4	4,1,1	0.615^a^
Disease duration	3.10 ± 1.45	3.67 ± 1.75	0.521^b^
CDR (0.5,1,2)	3,5,2	0,4,2	0.324^a^
MMSE	20.20 ± 5.11	18.33 ± 5.75	0.510^b^
ADAS-cog	25.62 ± 11.31	28.17 ± 7.15	0.630^b^
ACE-III	59.11 ± 21.51	49.33 ± 15.46	0.357^b^
AVLT	29.33 ± 7.37	24.80 ± 6.98	0.284^b^
ADL	28.20 ± 6.12	29.50 ± 3.94	0.651^b^

Results are expressed as mean ± standard deviation. rTMS-CT, rTMS combined with cognitive training; CDR, Clinical Dementia Rating scale; MMSE, Mini-Mental State Examination; ADAS-cog, Alzheimer's Disease Assessment Scale-Cognitive Subscale; ACE-III, Addenbrooke's Cognitive Examination III; AVLT, auditory verbal learning test; ADL, Activities of daily living. ^a^*p*-value obtained using Fisher's exact test. ^b^*p*-value obtained using a two-sample two-tailed Student's *t*-test.

### Effect of rTMS combined with cognitive training on neuropsychological assessments

**Figure 1:**
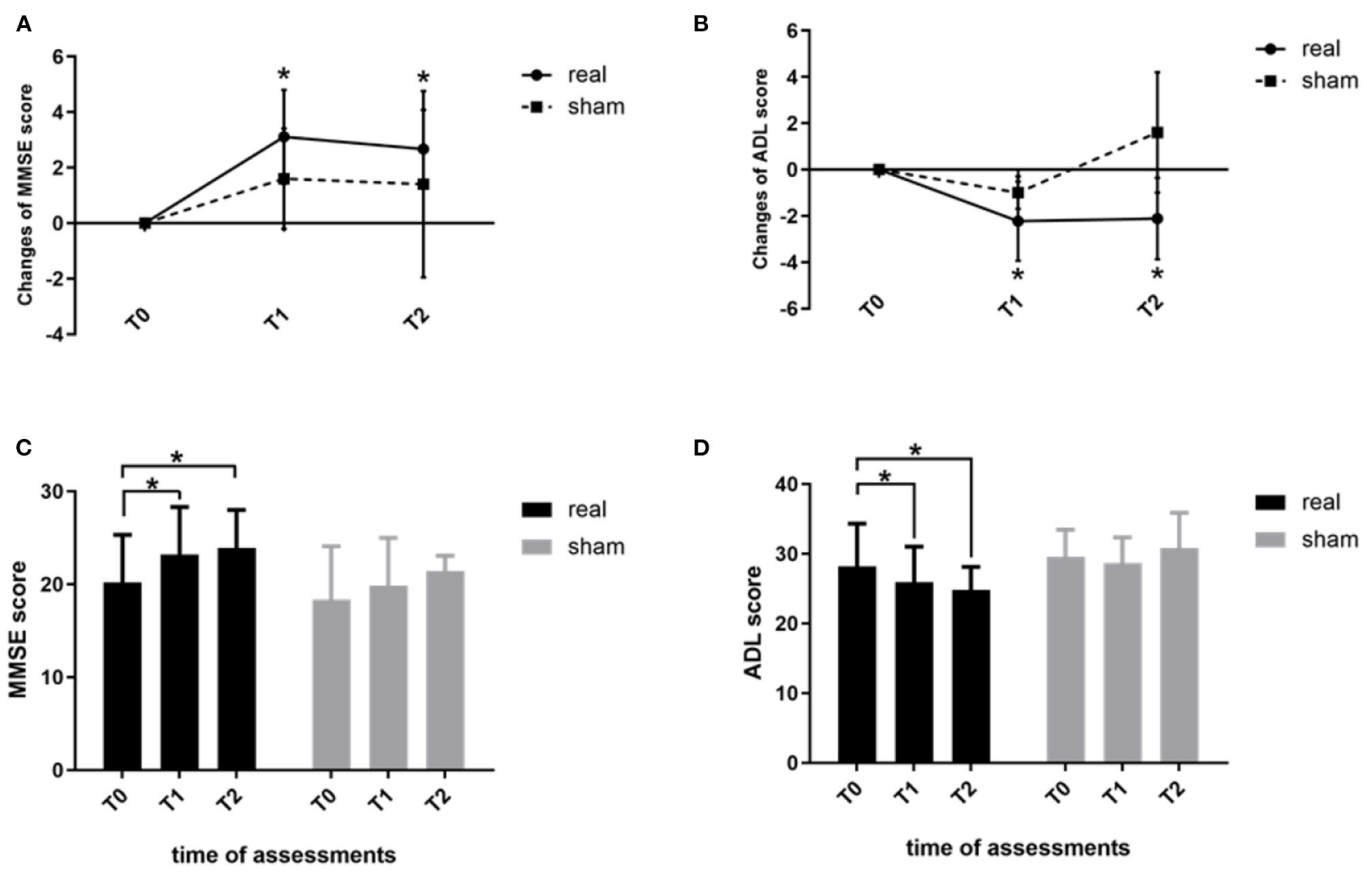
Changes in the MMSE **(A)** and ADL **(B)** scores immediately after treatment (T1) and at 4 weeks after treatment (T2) from baseline measurements (T0). **(C)** shows MMSE scores and **(D)** shows ADL score within the two groups at the three time points (T0, T1, T2). MMSE, Mini-Mental State Examination; ADL, activities of daily living. *p < 0.05. Error bars indicate SD.

Analysis of the effects of rTMS-COG therapy on neuropsychological assessments revealed significant group × time interactions for the MMSE (F = 5.339, *p* = 0.023, η^2^ = 0.433; Figure 1A) and ADL (F = 5.409, *p* = 0.039, η^2^ = 0.436; Figure 1B) scores. No other significant results were observed for the remaining test scores (*p* > 0.05). *Post-hoc* analysis indicated that the MMSE score significantly increased at the end of the 4 weeks' treatment (T1, *p* = 0.001) and 4 weeks after treatment (T2, *p* = 0.000) compared with baseline scores (Figure 1C), indicating that rTMS-COG therapy had both immediate and long-term effects on cognitive function improvement. ADL scores significantly decreased at T1 (*p* = 0.001) and T2 (*p* = 0.034) compared with baseline scores (Figure 1D), indicating better function after real rTMS-COG therapy. In contrast, the MMSE and ADL scores of the sham group remained relatively stable at the two timepoints after treatment (MMSE score: *p* = 0.337 at T1, *p* = 0.902 at T2; ADL score: *p* = 0.613 at T1, *p* = 0.207 at T2).

### Effect of rTMS combined with cognitive training on regional CBF

**Figure 2:**
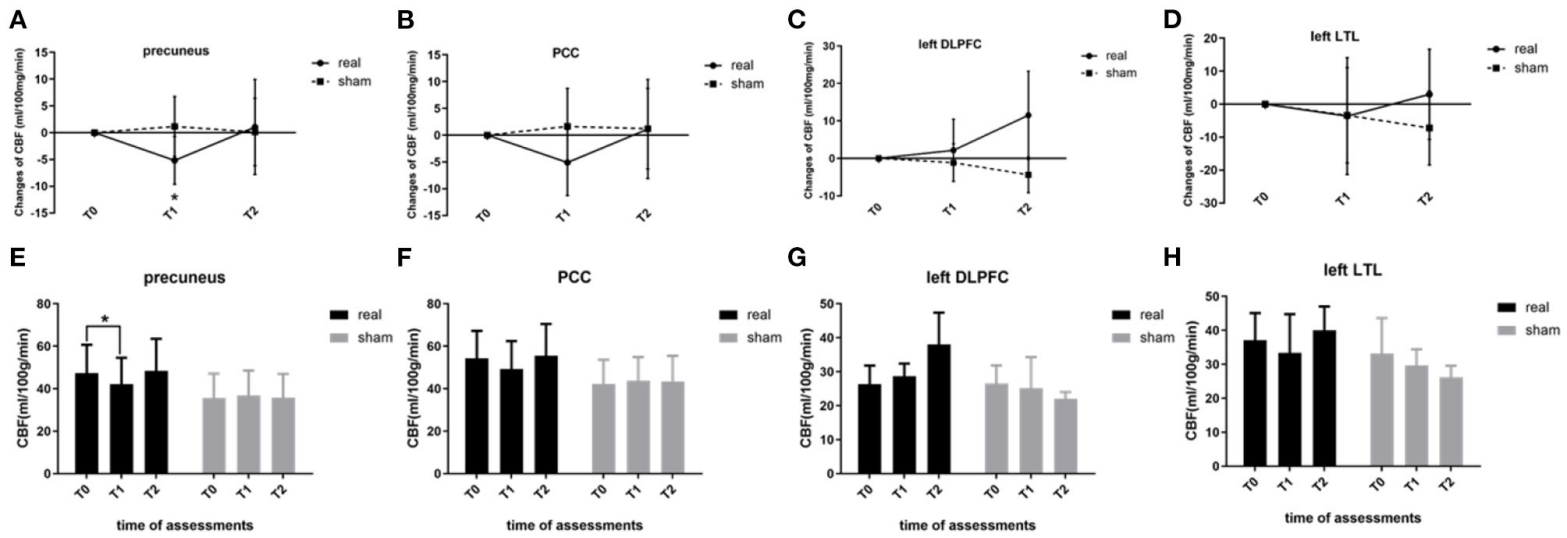
Regional CBF changes at the ROI level. **(A)** Precuneus, **(B)** PCC, **(C)** left DLPFC, and **(D)** left LTL. **(E–H)** show regional CBF within the two groups (real and sham) at three timepoints (T0, T1, T2). CBF, cerebral blood flow; ROI, region-of-interest; PCC, posterior cingulate cortex; DLPFC, dorsal lateral prefrontal cortex; LTL, lateral temporal lobe. *p < 0.05. Error bars indicate SD.

For ROI-level analysis, segmentation of the precuneus and PCC, as well as the stimulation targets (left DLPFC and left LTL), was checked visually, and image quality was rated as excellent for all participants. At baseline, the regional CBF values of the precuneus, PCC, and stimulation targets showed no significant difference between the two groups (real and sham, *p* > 0.05). As for the CBF changes induced by rTMS-COG therapy, the regional CBF of the precuneus showed a significant group × time interaction (F = 5.833, *p* = 0.027, η^2^ = 0.593; Figure 2A). CBF changes in the PCC and stimulation targets (left DLPFC and left LTL) showed no significant main or interaction effects (*p* > 0.05; Figures 2B–D). The regional CBF value of the precuneus in the real rTMS-COG group significantly decreased immediately after the end of 4 weeks' treatment (T1, *p* = 0.007) and returned to baseline 4 weeks after treatment (T2, p=0.783; Figure 2E). The sham group did not show significant CBF changes in the precuneus at the two timepoints (*p* = 0.629 at T1, *p* = 0.898 at T2). Although not statistically significant, the variation tendency of regional CBF in the PCC was much more similar to that in the precuneus but different from the stimulation targets (Figures 2F–H).

**Figure 3:**
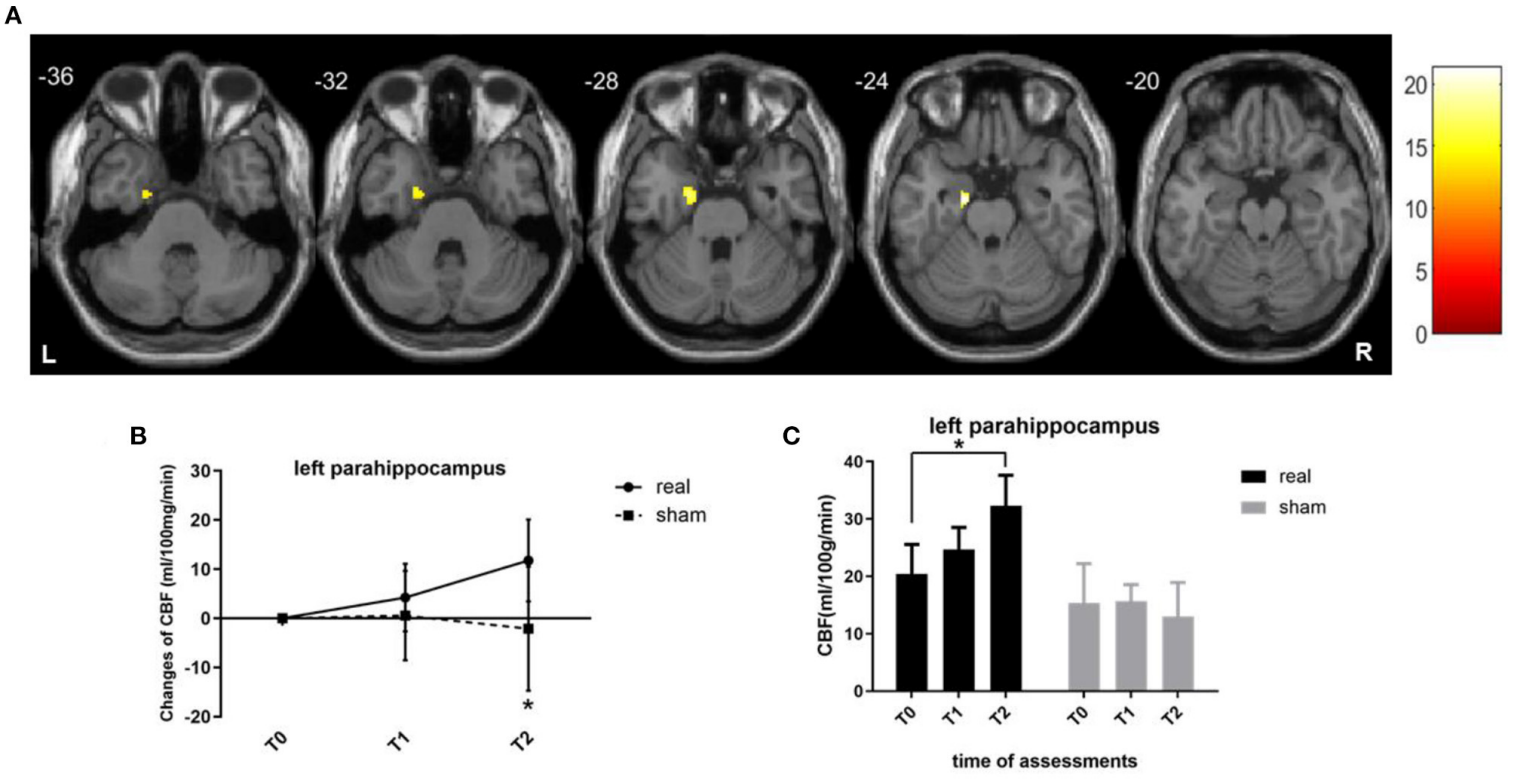
CBF changes at the voxel level. **(A)** Brain regions where regional CBF demonstrated treatment main effect. Brain images shown at an uncorrected threshold of *p* < 0.001, and slice numbers are in the MNI space. **(B)** Line graphs showing the regional CBF changes in the cluster of left parahippocampus. **(C)** Bar graphs showing the mean CBF values in the cluster of the left parahippocampus for the real rTMS (in black) and sham (in gray) groups. CBF, cerebral blood flow; MNI, Montreal Neurologic Institute. *p < 0.05. Error bars indicate SD.

For voxel-level analysis, a significant group main effect was detected in the left limbic lobe cluster, with the maximal peak in the left parahippocampus (*p* < 0.001, uncorrected, peak at [−16 −8 −24]; Figure 3A). Simple effect analysis indicated that the regional CBF of the left parahippocampus in the real group remained unchanged at T1 (*p* = 0.187) but significantly increased at T2 (*p* = 0.008), indicating a long-term effect on the regional CBF of the left parahippocampus (Figures 3B, C). The sham group showed non-significant CBF changes in the left parahippocampus at two timepoints (*p* = 0.757 at T1, *p* = 0.654 at T2). However, this did not survive family-wise error correction (FWE) based on cluster extent with a critical corrected alpha of 0.05 (pFWE <0.05). The variation tendency of CBF in the left parahippocampus was similar to that of the stimulation targets (left DLPFC), regardless of significance.

### Association between regional CBF changes and neuropsychological alterations

Exploratory analyses of the association between CBF changes and neuropsychological measurements indicated that CBF decrease in the precuneus correlated with a significant ACT-III score increase at the end of 4 weeks' treatment (T1; *r* = −0.732, *p* = 0.025), indicating that regional CBF decrease in the precuneus immediately after rTMS-COG therapy was associated with better cognitive states. CBF changes in the cluster of the left parahippocampus showed no significant correlation with any neuropsychological score alterations (*p* > 0.05).

## Discussion

This study provides further evidence of the beneficial effect of high-frequency rTMS combined with cognitive training (rTMS-COG) in patients with mild-to-moderate AD. Treatment-by-time interactions were observed on the neuropsychological measurements, showing significant improvement in cognitive function and ADL immediately after rTMS-COG therapy (T1) and 4 weeks after the therapy (T2). This may be a possible synergistic lasting after-treatment effect of rTMS-COG therapy, which has been supported by previous studies (17, 23, 40). For rTMS-COG-induced CBF changes, significant treatment-by-time interactions on the precuneus and the main effect of treatment on the left parahippocampus were noted, indicating immediate effects in the precuneus and long-term effects in the left parahippocampus. The CBF decrease in the precuneus immediately after rTMS-COG therapy correlated well with improved cognitive function.

### Synergistic effect of rTMS and cognitive training

Although the cognitive training tasks were the same in the real rTMS and sham groups, we still could not ascribe the improvement in clinical outcomes in the real group to rTMS itself. Therefore, rTMS may have a synergistic effect with active and passive cognitive training (41). By modulating neuroplasticity, rTMS might serve to “prime” a given targeted cortical region or network to make it more susceptible to the intervention of cognitive training (35). rTMS combined with cognitive training (rTMS-COG) is a useful adjuvant therapy for patients with AD that enhances cognitive function (3, 4, 41, 42). Interlaced with cognitive training (GOG), rTMS has additional beneficial effects, possibly as good as cholinesterase inhibitors (17).

### rTMS-COG induced immediate CBF reduction on the precuneus and was associated with cognition improvement

Compared with the sham group, CBF of the precuneus in the real rTMS group decreased significantly immediately after treatment (T1) and returned to baseline 4 weeks after treatment (T2). Although there was no significant main or interaction effect, CBF changes in the PCC showed a similar variation tendency as that in the precuneus. Our study revealed, for the first time, that rTMS-COG therapy induced CBF reduction in the precuneus of patients with AD immediately after treatment. AD has been assumed to be a disconnection syndrome associated with changes in neurocircuitry (43). The PCC/precuneus is widely connected to other areas of the brain and forms a hub node in the DMN (31). Both aging and AD are characterized by reduced DMN connectivity. However, the importance of pathological changes in the precuneus in AD is underscored by the fact that diminished connection of the DMN with the precuneus acts as a marker of AD pathology but not of aging (44). The precuneus connects extensively with regions of the brain involved in execution and memory retrieval functions, offering numerous targeting opportunities. Koch et al. (20) stimulated the precuneus in prodromal AD to enhance memory and neural activity. rTMS therapy targeting the precuneus improves posterior hippocampal connectivity and controls subjective cognitive decline, which is a potential preclinical stage of AD (21). The precuneus may be an effective therapeutic site for improving memory in AD (3). Targeting the left DLPFC, rTMS has also been reported to enhance the amplitude of low-frequency fluctuations and modulate the resting activity of the precuneus (18). Considering the above, it is expected that CBF changes in the precuneus in our study reflect its key role in maintaining DMN connectivity and signal transmission.

Importantly, we found that CBF decrease in the precuneus was associated with a significant increase in ACE-III scores at the end of 4 weeks' treatment (T1), indicating that CBF reduction in the precuneus immediately after rTMS-COG therapy was associated with better cognitive function. This is contradictory to previous findings because hypoperfusion of the PCC/precuneus was reported to correlate with cognitive decline measured by MMSE (45, 46). Because the neuropsychological benefits do not diminish 4 weeks after treatment, the regional CBF decrease in the precuneus might represent a transitory plastic stage in our study. In addition, the different tools used for neuropsychological assessment may have impacted the results, as CBF changes in the precuneus were only correlated with ACE-III scores. Comparing five cognitive instruments, including MMSE, for the diagnosis of AD, ACE-III achieved the highest diagnostic accuracy in a previous study (47). Our study may indicate that ACE-III can be used to detect rTMS-COG therapy-induced changes.

The reasons for reduced perfusion in the precuneus following rTMS-COG therapy in the present study are unclear. rTMS can modify brain cortical excitability and regional CBF, which is hemisphere- and frequency-dependent (25). rCBF decrease has been reported in patients with severe enduring anorexia nervosa, in which a significant reduction in amygdala CBF was observed after high-frequency rTMS on the left DLPFC (48). Measurement of CBF using ^99m^Tc HMPAO uptake on SPECT also showed that high-frequency TMS could decrease perfusion in remote areas of the brain in patients with drug-resistant depression (49). A decrease in the resting-state activity of the precuneus was accompanied by elevated levels of peripheral brain-derived neurotrophic factor (BDNF) following TMS stimulation in patients with AD (22). The BDNF genotype has been reported to affect anxiety-related personality traits and resting CBF in the frontolimbic neurocircuitry of healthy people (50). Whether peripheral BDNF plays a role in rTMS-COG therapy by influencing regional CBF requires further investigation.

### rTMS-COG induced weak long-term CBF increase on left parahippocampus

Whole-brain voxel-wise analysis revealed a main effect of treatment on the left parahippocampus with an uncorrected threshold of *p* < 0.001, which did not survive multiple comparison correction (pFWE <0.05). This result indicated that rTMS-COG therapy induced a weak CBF increase in the left parahippocampus 4 weeks after treatment (T2), showing a long-term effect in remote areas by stimulation of the left frontal and temporal lobes. Increases in rCBF usually suggest a cellular and vascular compensatory process (27). Although the effect of TMS-COG therapy on regional CBF cannot be ascribed to rTMS alone, our study still indicated that stimulation of the left DLPFC and left LTL induced rCBF changes involving the areas of frontolimbic circuits, which confirms the ability of rTMS to act on functionally and anatomically connected areas (5, 21, 51, 52).

### Limitations

This study had several limitations, including the small sample size. Therefore, although with no statistical significance, we could not conclude that rTMS-COG therapy induces no CBF changes in the brain stimulation targets/PCC. Additionally, the unequal sample sizes in the two groups (real and sham) and heterogeneity of patients enrolled reduced the statistical power. Because this is an exploratory neuroimaging study, future studies with a large sample size are needed to reveal the neurobiological basis of rTMS. Second, although an optical navigation system was used to locate the stimulation site, an individualized MRI navigation system is recommended to improve the accuracy of placement of the stimulation coil. With the development of hardware and computational science, multimodal neuroimaging data can be collected and imported into the navigation system to build a personalized brain model and personalized stimulation protocols (53). Third, it is difficult to attribute the observed cognitive changes to stimulation alone because we did not include a third group that received rTMS alone. As mentioned previously, our study reflects the synergistic effect of rTMS and cognitive training. Finally, additional measurements such as task fMRI or resting-state fMRI were not conducted to further clarify what exactly happened at the functional connectivity level. Because the effects of rTMS are complex and involve multiple neurotransmission systems, regions, and networks (54), further studies combining TMS with imaging studies would advance our understanding of the neurobiological underpinnings of rTMS, either using positron emission tomography (PET) or MR techniques.

## Conclusion

The study provides further evidence that high-frequency rTMS-COG produces beneficial immediate and long-term effects in patients with AD regarding cognitive function and ADL. rTMS targeting the left DLPFC and left LTL combined with cognitive training induced decreased CBF in the precuneus immediately after treatment and increased CBF in the left parahippocampus 4 weeks after treatment in patients with mild-to-moderate AD, showing a region-dependent alteration. The results of our study provide imaging evidence to understand the underlying neurobiological mechanisms for the application of rTMS-COG in AD.

## Data availability statement

The raw data supporting the conclusions of this article will be made available by the authors, without undue reservation.

## Ethics statement

The studies involving human participants were reviewed and approved by the Ethics Committee of Tongji Hospital (Wuhan, China). The patients/participants provided their written informed consent to participate in this study.

## Author contributions

YQ: conceptualization, methodology, and software. YQ and LB: data curation and writing—original draft preparation. FZ: visualization and investigation. SJ: software. MZ: supervision and validation. WZ: writing—reviewing and editing. All authors contributed to the article and approved the submitted version.
